# A *Chlamydia trachomatis* strain with a chemically generated amino acid substitution (P370L) in the *cthtrA* gene shows reduced elementary body production

**DOI:** 10.1186/s12866-015-0533-2

**Published:** 2015-09-30

**Authors:** James W. Marsh, Bryan A. Wee, Joel D.A. Tyndall, William B. Lott, Robert J. Bastidas, Harlan D. Caldwell, Raphael H. Valdivia, L. Kari, Wilhelmina M. Huston

**Affiliations:** Institute of Health and Biomedical Innovation (IHBI), Queensland University of Technology (QUT), 60 Musk Avenue, Kelvin Grove, QLD 4059 Australia; National School of Pharmacy, University of Otago, PO Box 56, Dunedin, 9054 New Zealand; Department of Molecular Genetics and Microbiology, Center for Microbial Pathogenesis, Duke University Medical Center, Durham, NC 27710 USA; Laboratory of Intracellular Parasites, National Institute of Allergy and Infectious Diseases, National Institutes of Health, Hamilton, MT 59840 USA

**Keywords:** Chlamydia, HtrA, Genetics, Heat shock, Intracellular

## Abstract

**Background:**

*Chlamydia* (*C.*) *trachomatis* is the most prevalent bacterial sexually transmitted infection worldwide and the leading cause of preventable blindness. Genetic approaches to investigate *C. trachomatis* have been only recently developed due to the organism’s intracellular developmental cycle. HtrA is a critical stress response serine protease and chaperone for many bacteria and in *C. trachomatis* has been previously shown to be important for heat stress and the replicative phase of development using a chemical inhibitor of the *Ct*HtrA activity. In this study, chemically-induced SNVs in the *cthtrA* gene that resulted in amino acid substitutions (A240V, G475E, and P370L) were identified and characterized.

**Methods:**

SNVs were initially biochemically characterized in vitro using recombinant protein techniques to confirm a functional impact on proteolysis. The *C. trachomatis* strains containing the SNVs with marked reductions in proteolysis were investigated in cell culture to identify phenotypes that could be linked to *Ct*HtrA function.

**Results:**

The strain harboring the SNV with the most marked impact on proteolysis (*cthtrA*_P370L_) was detected to have a significant reduction in the production of infectious elementary bodies.

**Conclusions:**

This provides genetic evidence that *Ct*HtrA is critical for the *C. trachomatis* developmental cycle.

**Electronic supplementary material:**

The online version of this article (doi:10.1186/s12866-015-0533-2) contains supplementary material, which is available to authorized users.

## Background

*Chlamydia trachomatis* is the most prevalent bacterial sexually transmitted infection worldwide and the leading cause of preventable blindness [1, 2]. If left untreated, chlamydial infection can lead to serious and costly conditions including infertility, ectopic pregnancy, epididymitis, and pelvic inflammatory disease. As intracellular bacteria, the *Chlamydiae* are defined by their unique development cycle where small, infectious elementary bodies (EB) invade a host cell and differentiate into large, non-infectious reticulate bodies (RB) that replicate by binary fission [3]. Due to the absence of several biosynthesis pathways that are common to most other bacteria, *Chlamydia* has a reduced genome size (~1 Mb) that requires the organism to rely on the host cell for nutrients and survival [4]. As a result, *Chlamydia*’s obligate intracellular development cycle had hampered progress towards genetic techniques and the organism’s pathogenic mechanisms have not been fully elucidated.

HtrA is a critical protease and chaperone for many bacteria and has been implicated in several functions including stress response, protein quality control, outer membrane protein (OMP) localization and assembly, host cell manipulation, and virulence [5–9]. The distinct biochemical functions of proteolysis and chaperone activity are mediated by the allosteric activation of the protein to oligomers ranging from trimers to hexamers to at least 24-mers [7, 10]. Using homology modeling and biochemical methods, we have previously suggested that *C. trachomatis* HtrA (*Ct*HtrA) proteolytic activation and oligomerization are mediated by distinct structural pathways, and that specificity of the substrate binding may differentiate protease and chaperone activity [11, 12]. HtrA is highly conserved in *Chlamydia* and the application of a chemical inhibitor of *Ct*HtrA protease activity indicated that *Ct*HtrA is important for the replicative phase of the chlamydial developmental cycle, important for the recovery from penicillin-induced chlamydial persistence, and has a critical role during heat stress [13–18]. These data, relying on both biochemical and inhibitor-based approaches, enabled significant insight into *Ct*HtrA function and the role of specific parts of the amino acid sequence for these functions, and provided the foundation for this attempt to use genetics to investigate *Ct*HtrA’s physiological role for *C. trachomatis*.

In the absence of genetic techniques such as targeted gene replacement and transposon-based mutagenesis, the field has instead relied on genomic, proteomic, and transcriptomic methods for chlamydial research. However, functional validation requires genetic evidence. In response, several molecular strategies have recently proven successful including small molecule inhibitors [15, 19], random chemical mutagenesis [20], lateral gene transfer [21], and shuttle vector transformations [22]. Given our existing data on the structure and function of *Ct*HtrA, genetic approaches such as random chemical mutagenesis are a promising approach to for the characterisation of *Ct*HtrA in *C. trachomatis*.

Chemical mutagenesis is the use of chemical compounds to increase the frequency of chromosomal mutations above the spontaneous rate [23]. The types of DNA lesions produced include DNA adducts, intercalation, inter-strand cross-linking, alkylation, and/or base modifications, which result in base-pair substitutions, frameshift mutations, and/or deletions in the nucleotide sequence. In 2011, Kari et al. presented the development of a reverse genetics approach using chemical mutagenesis to generate isogenic mutants of *C. trachomatis* [24], demonstrating that EMS is a viable mutagen for genetic studies in *Chlamydia*. EMS is an alkylating agent that specifically acts at nitrogen positions in nucleotide bases to produce GC to AT transition mutations [25]; based on the GC content of the *C. trachomatis* L2 434/Bu genome, there are 429,520 potential sites that could theoretically be mutated by EMS. Thus, single nucleotide variants (SNV), induced by the EMS treatment of *C. trachomatis*, can be identified in a gene of interest and subjected to a genetic characterization of its function. Given our awareness of the residues and structural elements that are important for *Ct*HtrA’s protease and chaperone activity [11, 12, 17, 18], we investigated several SNVs in the *cthtrA* gene to examine the protein’s physiological function for *C. trachomatis.*

## Methods

### Chlamydial culture conditions

*Chlamydia trachomatis* was routinely cultured in McCoy B or HEp-2 cells in the presence of DMEM supplemented with 10 % fetal calf serum (FCS) at 37 °C, 5 % CO_2_. Experiments were typically conducted at an MOI of 0.3. For the calculation of infectious yield, cultures harvested in SPG buffer (10 mM sodium phosphate, 250 mM sucrose, 5 mM glutamic acid, pH 7.4) were serially diluted and cultured in fresh McCoy B cell monolayers until 30 h post-infection, when they were fixed with methanol and stained for microscopy. The infectious yield was determined by the number of inclusion forming units from each milliliter of the original culture.

### Chemical mutagenesis

Chemical mutagenesis of *C. trachomatis* L2 was performed by the Valdivia Laboratory at Duke University, NC, USA [21]. Vero cells were infected with Rif^R^*C. trachomatis* serovar LGV biovar L2 434/Bu at an MOI of 5.0 for 18 h and exposed to 20 mg/mL ethyl methanesulfonate (EMS) in PBS for 1 h, as described [21]. EMS was prepared in phosphate buffered saline (PBS) with 0.9 mM calcium chloride and 0.49 mM magnesium chloride [26]. The cells were washed three times in PBS + CaCl_2_/MgCl_2_ to remove residual mutagen and incubated in DMEM/5 % FCS at 37 °C in a 5 % CO_2_ humidified incubator for 48–72 h to allow the bacteria to recover and complete their developmental cycle. EBs were harvested by hypotonic lysis of infected cells with sterile water, followed by addition of 5× SPG media to a final concentration of 1× SPG, titered for IFUs, plaque-purified as described [21] and stored at−80 °C. The frequency of mutagenesis was determined by inducing rifampicin resistance, followed by the plaque purification using 7 × 10-fold serial dilutions of bacteria in the presence of 200 ng/mL rifampicin [26]. The frequency of rifampicin resistance was defined as the number of rifampicin-resistant plaques divided by the total number of bacteria plated [26]. *C. trachomatis* L2 chlamydial mutant strains containing SNVs in *cthtrA* were identified from whole genome sequences generated from a collection of 934 chemically mutagenized *C. trachomatis* L2 strains (sourced from the Valdivia laboratory for this study, investigated in the Huston Lab in Australia). Isolates from this collection containing *cthtrA* variants were further purified by plaque isolation (in Australia).

*C. trachomatis* D was mutagenized four consecutive times in the Caldwell Laboratory, NIAID Rocky Mountain Laboratories, Montana. At each round, infected McCoy cells were exposed to 5–7 mg/mL EMS 19 h post-infection for 1 h and chlamydiae were harvested 40–44 h post-infection. A library of sub-populations were built to screen for *cthtrA* mutants. McCoy B cells in 96-well tissue culture plates were infected with 8 inclusion-forming units per well. Chlamydiae were harvested at 48 h post-infection and were used to reinfect McCoy B cells in 96-well tissue culture plates. Infected cells were harvested and passaged for a third time, and DNA extracted from the third passage was used to PCR amplify *cthtrA*. Amplicons were heat denatured, slowly reannealed, and digested by CEL I endonuclease. Digestion products were visualized by DNA agarose gel electrophoresis. Mutations detected by CEL I digestion were identified by Sanger sequencing (these strains and this work to identify the mutations was conducted in the Caldwell laboratory, characterization of the strains was conducted in the Huston laboratory).

### In silico analysis of cthtrA SNVs

*Ct*HtrA sequence alignments, 2D structural motifs, and homology models for the hexameric and 24-meric oligomers were used for the characterisation of EMS mutants, as previously described [11]. Each mutation was examined in both oligomeric models of *Ct*HtrA with the PyMOL Molecular Graphics System, Version 1.7.4 Schrödinger, LLC, according to secondary structure localisation and potential impact on *Ct*HtrA function.

### Site-directed mutagenesis and recombinant protein expression

The previously generated *Ct*HtrA recombinant expression construct was used for this study [17]. Site-directed mutagenesis was used to introduce point mutations corresponding to the EMS mutations identified in the chlamydial strains using a QuikChange II XL Site-Directed Mutagenesis Kit (Stratagene) using the primers A240Vsdm, E47Ksdm, G268Rsdm, G475Esdm, P370Lsdm, and R55Qsdm (Additional file 1: Table S4). All mutations were confirmed by Sanger sequencing. Heterologous recombinant protein expression and purification of *Ct*HtrA recombinant mutated proteins were completed as previously published [11, 12, 17].

### Protease assays

Full-length β-casein cleavage assays were initially used to test the proteolytic activity of wild type *Ct*HtrA and the mutants. Up to 2 mg of recombinant *Ct*HtrA was incubated with 10 mg of β-casein in 50 mM Tris, 20 mM MgCl_2_ and examined with SDS-PAGE as previously described [17]. The gel bands were quantified by densitometry using a Li-Cor Odyssey 9120 Infrared Imaging System. The rate of proteolysis was tested with a peptide substrate labelled with 7-methoxycoumarin-4-acetic acid (MCA; fluorophore) and 2,4-dinitrophenyl (DNP; quencher) in black plates at 37 °C using a POLARstar Optima D77656 fluorimeter. Substrate specificity was examined using peptides labelled with *para*-Nitroanilide (*p*NA) that were incubated with *Ct*HtrA at 37 °C and analysed with an xMARK microplate spectrophotometer. All protein and peptide substrates and allosteric activators are listed in Additional file 1: Table S5. All peptides and activators were synthesised by Mimotopes (Melbourne, VIC, Australia) to 95 % purity and were resuspended in 50 % isopropanol or DMSO. Statistical analyses were conducted using an unpaired *t*-test, calculated with Prism software. All assays were conducted on two separate occasions, in triplicate.

### Oligomerization assays

The ability of *Ct*HtrA or the mutants to oligomerize to higher oligomers was examined by crosslinking oligomers with glutaraldehyde as previously described [12]. The samples were separated on 3–8 % Tris-Acetate gradient polyacrylamide gels and examined by silver staining. All assays were conducted in triplicate on three separate occasions. HiMark pre-stained molecular weight marker (Life Technologies, USA) was included on each gel.

### Heat shock of chlamydial culture

Heat shock assays were performed by infecting HEp-2 cells in a 48-well plate with *Ct*L2_wt_ and each mutant at an MOI of 0.3 in DMEM at 37 °C, 5 % CO_2_. Cultures were heat shocked for 4 h at 42 °C, 5 % CO_2_ beginning at 20 h post-infection, before returning the cultures to 37 °C for the remainder of the development cycle, prior to the measurement of infectious progeny [14].

### Chlamydia growth curves

Growth curve assays were performed by infecting McCoy B cells in a 96-well plate with *Ct*L2_wt_ and each of the mutants at an MOI of 0.3. The cells were incubated in DMEM at 37 °C, 5 % CO_2_ for up to 48 h. At each time point the cultures were harvested by media removal, the addition 200 μL SPG, and storage at −80 °C. The plates were thawed and the McCoy B cells lysed by pipette disruption and cell lysates for each time point were serially diluted and used to re-infect fresh McCoy B cell monolayers in DMEM + 1 μg/mL cycloheximide on 96-well plates, in triplicate. The infectious yield was determined as described above.

### Morphological analysis and immunocytochemistry

*Chlamydia* cultures were visualised with immunofluorescence as previously described [16, 27]. Briefly, confluent McCoy B cells seeded on 8 mm coverslips in a 48-well plate were infected with *Chlamydia* at an MOI of 0.3 in DMEM media and were stained with the *Chlamydia* Cel LPS product (Cellabs, Brookvale, NSW, Australia) as per the manufacturer’s instructions. Primary antibody was incubated for 1 h in 1 % BSA followed by two washes in PBS. Secondary antibodies were Alexa Fluor 488 goat anti-rabbit (Life Technologies, USA) and were used at 1/600 dilution in 1 % BSA for 1 h. Coverslips were mounted using Prolong Gold antifade (Life Technologies, USA) and viewed under a Leica TCS SP5 confocal laser scanning microscope (Leica Microsystems). Images were captured using the Leica application suite for advanced fluorescence.

### Genetic analysis of C. trachomatis strains with EMS generated mutations

*Ct*L2_wt_ and mutants were semi-purified and genomic DNA was extracted using the phenol-chloroform method as previously described [28]. Whole genome sequencing was performed commercially by the Micromon sequencing facility on the Illumina MiSeq platform, using theTruSeq v3 chemistry and the NexteraXT Library Preparation Kit (Monash University, Victoria, Australia). SNV analysis was performed using Nesoni (https://github.com/Victorian-Bioinformatics-Consortium/nesoni) with the *C. trachomatis* L2 434/Bu complete genome (RefSeq accession: NC_010287) serving as the reference sequence.

### Lateral gene transfer

Spectinomycin-resistant (Spc^R^) *C. trachomatis* L2 isolates and rifampicin-resistant (Rif^R^) mutant isolates were generated by repeated passage on McCoy B cells in the presence of sub-inhibitory concentrations of the antibiotic. Passaging continued until maximum resistance was reached in the presence of 200 ng/mL rifampicin or 200 μg/mL spectinomycin. Lateral gene transfer between the Spc^R^*Ct*L2_wt_ and Rif^R^ mutant was induced by co-infecting each strain at an MOI of 4.0 in 12 wells of a 24-well plate (total MOI of 8.0). Positive controls were included by infecting duplicate wells in the presence of rifampicin only or spectinomycin only. Negative control wells with no antibiotics were also included. The cell lysates from each well were serially diluted and used to infect a fresh McCoy B cell monolayer on 6-well plates for plaque purification cultures [21].

## Results

### Chemical mutagenesis allows identification of SNVs that impact on CtHtrA function

Two existing libraries of *C. trachomatis* mutants were screened for mutations in the *cthtrA* gene, one based on *C. trachomatis* serovar L2 (CTL0195) and the other on *C. trachomatis* serovar D (CT_823). The screening of two libraries was performed to enable enable higher coverage of the *cthtrA* gene, while enabling the reduction of possible SNV bias given that the libraries were prepared using different methods. The *C. trachomatis* L2 and D serovars share 99.5 % nucleotide identity and 99.4 % amino acid identity at the *cthtrA* locus and thus are not expected to exhibit functional differences.

Firstly, we determined that there were 797 C/G to T/A mutations possible in *cthtrA*; 601 would be non-synonymous, 160 synonymous, and 36 nonsense. We have previously used a chemical inhibition approach to demonstrate that *Ct*HtrA is critical for the survival of *C. trachomatis* [15], so it was expected that strains harboring null mutations in *cthtrA* would be non-viable. However, strains containing *cthtrA* alleles with non-synonymous mutations in functionally important residues could potentially be used to investigate *Ct*HtrA physiological function(s). The first library screened was a collection of 937 *C. trachomatis* serovar L2 (434/Bu) mutant strains in which each SNV was mapped by whole genome sequencing. This library was generated with a mutation frequency ranging from 6 to 25 mutations per genome following treatment with EMS and ENU [29]. The second library screened was based on *C. trachomatis* serovar D-LC (*Ct*D-LC) [30] and included ~4600 mutant strains with a mutation frequency of seven SNVs per genome. Between the two libraries a total of 5537 mutants were screened and a total of 52 *cthtrA* variants were identified (Additional file 1: Table S1). Eighteen variants had synonymous substitutions and were excluded from further analysis, while the remaining 35 were analyzed *in silico* to predict their functional impact based on our previous *in silico* and biochemical understanding of the *Ct*HtrA amino acid sequence and associated functions [11, 12].

### Three CtHtrA mutations have an impact on in vitro proteolysis but not oligomerization

**Fig. 1:**
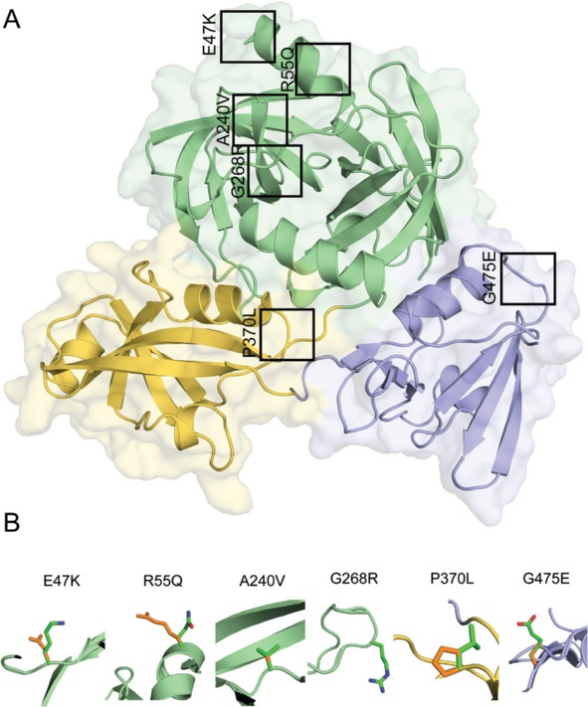
Mutated residues shown on the *Ct*HtrA monomer of the active 24-meric structure. **a**. The *Ct*HtrA active monomer with black boxes indicating the location of mutated residues. Green: protease domain, orange: PDZ1 domain, blue: PDZ2 domain. **b**. Close-up of wild-type residues (*orange side-chain*) aligned with the mutated residue (*green side-chain*)

The *cthtrA* EMS mutations were examined using molecular models of the inactive hexamer and active 24-mer oligomers of *Ct*HtrA, according to 2D and 3D structural motifs known to have a role in *Ct*HtrA protein function [11, 12, 17, 18]. Mutations were identified throughout the entire *cthtrA* gene, with SNVs identified in the signal peptide, protease domain, PDZ1 domain, and PDZ2 domain (Additional file 1: Table S1). Six mutations that resulted in amino acid substitutions were selected for characterization based on *in silico* prediction of a structural and/or functional impact: E47K (G – A), R55Q (G – A), A240V (C – T), G268R (G – A), P370L (C – T), and G475E (G – A) (Fig. 1a). The E47K and R55Q mutations were located at the N-terminus of the protease domain, upstream from loop LA, and had the potential to disrupt the trimeric protease domain interface by forming a steric clash with an adjacent protease domain loop (Fig. 1b). A240V represented a minor substitution from alanine to valine, two small and hydrophobic amino acids, however this residue is situated on loop L1 near to the catalytic serine and any conformational shift that may occur as a result of this mutation, however minor, would likely impact *Ct*HtrA proteolytic activity (Fig. 1b). G268R was situated in the protease domain active site in loop L2 and potentially results in a steric clash with loop L2 from an adjacent protease domain, also potentially disrupting proteolysis (Fig. 1b). P370L was located in the PDZ1 domain at the base of the ‘carboxylate binding loop’ and is likely to disrupt the conformational turn of this loop, which may impact the binding of the substrate C-terminus to the PDZ1 domain and the subsequent activation cascade (Fig. 1b). G475E was located in the PDZ2 domain and resulted in a potential steric clash with a nearby PDZ2 domain loop, as well as an adjacent PDZ1 domain in the oligomeric state, thus potentially affecting the oligomerization mechanism (Fig. 1b). These six mutations were then generated in vitro in our *Ct*HtrA recombinant protein expression construct using site-directed mutagenesis for biochemical characterization of the recombinant mutated proteins.

**Fig. 2:**
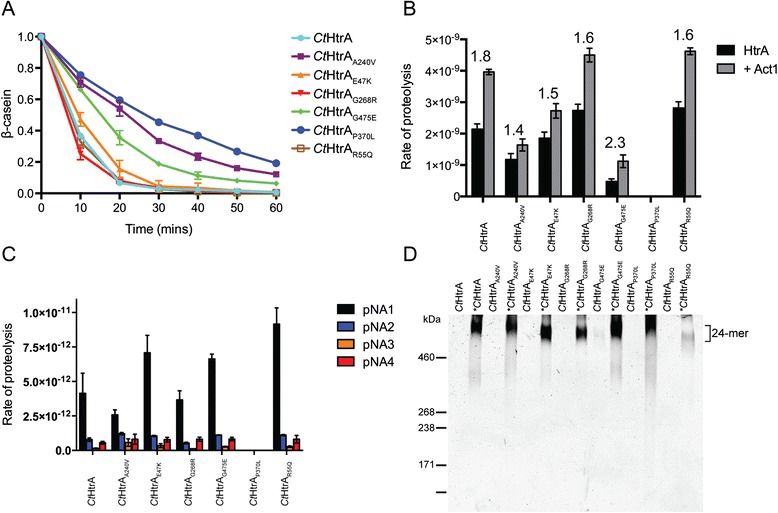
In vitro biochemical activity of wild type *C. trachomatis* L2 and recombinant mutants. **a.** Cleavage of full-length β-casein by wild type *Ct*HtrA and recombinant mutants over a 60 min time course. The corresponding SDS-PAGE gels are provided in Additional file 2: Figure S1. **b.** Rate of proteolysis of the βcas1 peptide substrate with and without allosteric activation (Act1 peptide) by recombinant proteins. Numbers above the bars represent the fold-change in proteolytic activity following the addition of the Act1 activator. Rate of proteolysis is measured as μM MCA min^−1^ μg *Ct*HtrA^−1^. Error bars represent standard error of the mean (*n* = 6). **c.** Measurement of the proteolytic activity of wild type *Ct*HtrA and mutants in the presence of pNA1-4 peptide substrates. Rate of proteolysis is measured as pNA 405 nm min^−1^. Error bars represent standard error of the mean (*n* = 6). **d.** Wild type *Ct*HtrA and the mutants oligomerise to 24-mer in the presence of full-length β-casein. The figure shows glutaraldehyde crosslinked protein samples prepared by oligomerisation assays following separation on 3–8 % TrisAcetate gels prior to silver staining. The size of the 24-meric oligomer is indicated to the right of the gel and the molecular weights are indicated on the left (High Molecular Weight Marker, Invitrogen)

The proteolysis and oligomerization activities of these recombinant proteins were compared to the wild-type recombinant protein (*Ct*HtrA). All recombinant proteins were able to cleave full-length β-casein at 37 °C, with proteins *Ct*HtrA_E47K_, *Ct*HtrA_G268R_, and *Ct*HtrA_R55Q_ displaying similar proteolytic activity to the wild-type (Fig. 2a). The rate of proteolysis was slower for the *Ct*HtrA_A240V_, and *Ct*HtrA_G475E_ proteins compared to the wild type, while the proteolysis activity of the *Ct*HtrA_P370L_ was substantially reduced (Fig. 2a; Additional file 2: Figure S1). Similarly, in the presence of a peptide substrate, the *Ct*HtrA_A240V_ and *Ct*HtrA_G475E_ proteins demonstrated a reduction in proteolytic rate, respectively, compared to the wild-type, while no proteolytic activity could be detected for the *Ct*HtrA_P370L_ protein (Fig. 2b). The proteolytic rate of the *Ct*HtrA_G268R_ and *Ct*HtrA_R55Q_ proteins were not substantially different from the wild-type. We have previously shown that the addition of a second peptide based on the 12 C-terminal residues of β-casein (Act1) can activate or increase the proteolytic activity of *Ct*HtrA [11, 12]. Thus, the activation of proteolysis by the recombinant proteins was investigated and a 1.4–2.3-fold increase in the rate of proteolysis was observed for *Ct*HtrA_E47K_, *Ct*HtrA_G268R_, *Ct*HtrA_R55Q_, *Ct*HtrA_A240V_ and *Ct*HtrA_G475E_. Alternatively, *Ct*HtrA_P370L_ displayed no detectable proteolytic activity against this peptide substrate (Fig. 2b), which may be due to this mutation causing a conformational change to the ‘PDZ1 activation cleft’, impacting the correct binding of Act1. Substrate specificity of the proteins was also examined using different *para-*Nitroanilide (pNA)-labeled substrates that differ in length and sequence (P1 – P4). No differences in substrate specificity were observed for any of the proteins compared to the wild type, suggesting that the mutations do not appreciably change the conformation of the catalytic domain (at least using these substrates; Fig. 2c). The *Ct*HtrA_P370L_ protein again displayed no proteolytic activity. *Ct*HtrA has been previously shown to oligomerize from a resting hexamer to an activated 12–/24-mer in the presence of both peptide and protein substrates [11, 12]. To investigate whether any of the recombinant mutated protein disrupted or impaired the activation of oligomerization, each were incubated with full-length β-casein at 37 °C and cross-linked with glutaraldehyde. In the presence of substrate, each mutant formed particles that are consistent with a higher order oligomer of CtHtrA (such as a 24-mer), with no evidence of the hexameric or trimeric forms that are observed when the oligomerization mechanism has been disrupted [12, 31]. While this method cannot detect differences in oligomeric structure or the exact number of monomers present, oligomeric activation to some form of oligomer appears to be unperturbed for each of the mutants (Fig. 2d) [11]. These data demonstrated that the *Ct*HtrA_A240V_, *Ct*HtrA_G475E_, and *Ct*HtrA_P370L_ mutations resulted in an appreciable disruption to the in vitro proteolytic activity of *Ct*HtrA and therefore isolates with these three mutations were selected from the libraries for further in vitro analysis of their phenotypic impact on chlamydial growth.

### A strain with a P370L substitution in cthtrA exhibits decreased or delayed EB production

**Fig. 3:**
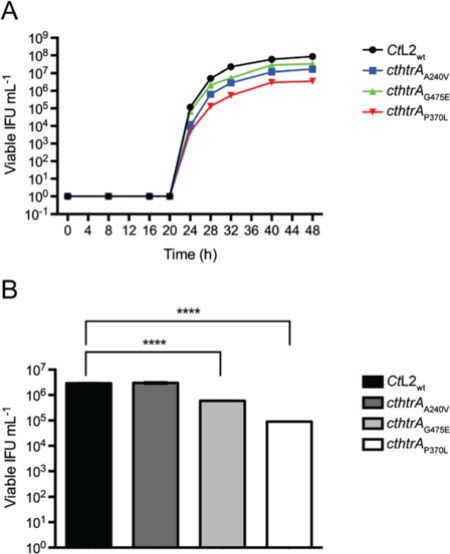
Growth and heat shock response phenotypes for *C. trachomatis* mutants containing *cthtrA* SNVs. a. One-step growth curve for *Ct*L2_wt_ and mutants over a 48 h time course. McCoy B cells were infected at an MOI of 0.3 and collected over the time course for determination of inclusion-forming units (IFUs). **b.** Infectious yield of *Ct*L2_wt_ and mutants at 44 h post infection after 4 h heat shock (42 °C, 5 % CO_2_; 20–24 h post infection). **** indicates *p* < 0.0001. Error bars represent standard error of the mean (*n* = 27)

Previous work by our group suggested that *Ct*HtrA has a crucial role in the replicative phase of *C. trachomatis* development, as the JO146 *Ct*HtrA inhibitor was lethal when added to the cultures at mid-replication [15]. Accordingly, growth rates were assessed for *Chlamydia* strains containing the *cthtrA* variants *cthtrA*_A240V_, *cthtrA*_P370L_, *cthtrA*_G475E_ and wild-type *C. trachomatis* serovar L2 (referred to as for this study *Ct*L2_wt_) (Additional file 1: Table S2), by determining the time at which infectious EBs were first detected, with additional times points during the growth cycle. The presence of infectious EBs was detected between 20 and 48 h post-infection, with each mutant strain producing fewer EBs than the wild-type (Fig. 3a). The *cthtrA*_P370L_ mutant produced significantly fewer infectious EBs compared to the wild type and other mutants, displaying a ~20–40-fold reduction (~95 % reduction) in infectious EB progeny (for example at 32 h post infection, 2.26 × 10^7^ EBs were detected for *Ct*L2_wt_ while 5.45 × 10^5^ EBs were detected for *cthtrA*_P370L_, corresponding to a 41.4-fold reduction in infectious EB production at this time point).

### The C. trachomatis isolate harboring a P370L substitution in cthtrA displays increased susceptibility to heat shock

The role of bacterial HtrA during heat shock has been widely reported [32–34], and *Ct*HtrA in particular has been shown to be upregulated during heat stress conditions [16] and critical for heat stress survival when the chemical inhibitor against *Ct*HtrA was used [14]. Therefore, *Chlamydia* strains with functionally disruptive mutations in *cthtrA* are likely to be more severely impacted by heat stress. HEp-2 cells were infected with each strain and subjected to 42 °C heat shock during the replicative phase (20 h post-infection) for 4 h, prior to restoration to 37 °C for the remainder of the development cycle. The impact of heat shock treatment on the mutants was determined by calculating the subsequent infectious yield from cultures harvested at 44 h post infection. The *cthtrA*_A240V_ mutant had an infectious yield that was comparable to the wild-type, while both the *cthtrA*_G475E_ and *cthtrA*_P370L_ mutants exhibited a significant reduction in infectious yield following heat shock, relative to the wild-type (Fig. 3). Notably, the *cthtrA*_P370L_ mutant resulted in a 32.5-fold reduction in infectious EB yield following heat shock compared to the wild type (*p* < 0.0001).

### The cthtrA_P370L_ inclusions are smaller in size

**Fig. 4:**
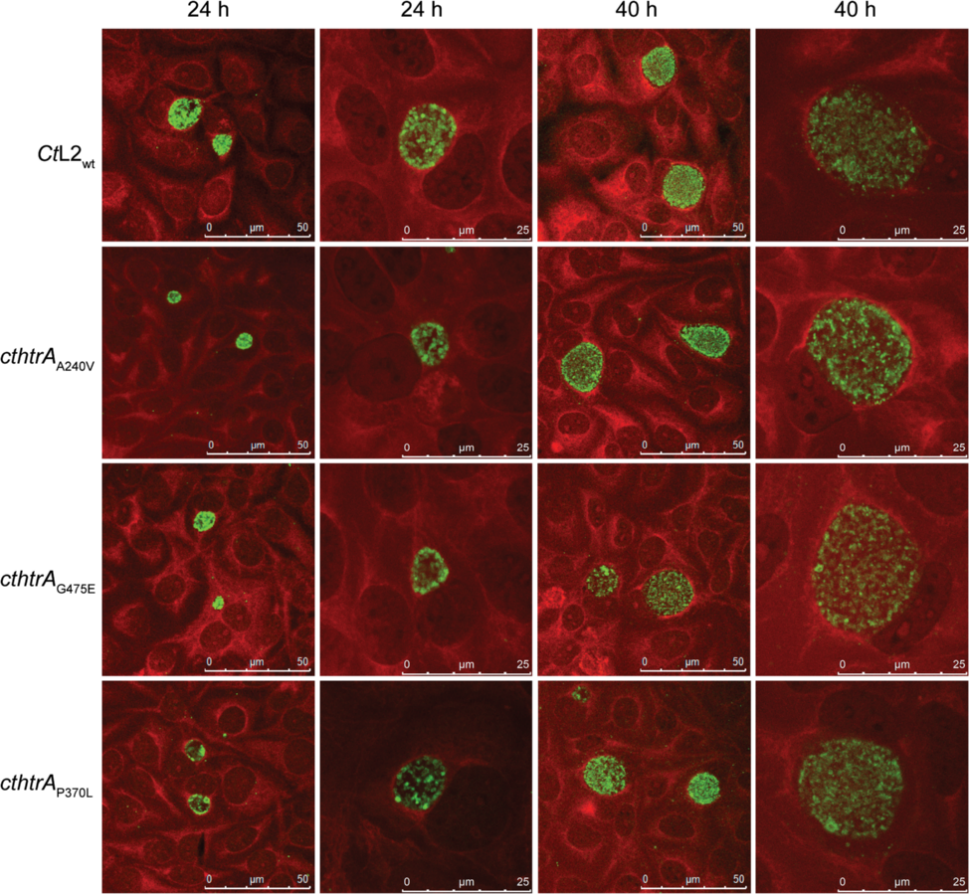
Confocal microscopy images of *Ct*L2_wt_ and mutants at 24 h and 44 h post infection. The *Chlamydia* isolates (*Ct*L2_wt_ and mutant) are shown to the left of the panels and the time point is shown above the panels. The second and fourth images for each isolate are enlarged representations of single inclusions. The image colours are, green: LPS (FITC anti-chlamydial LPS); red: host cells (Evans blue). The scale bars (bottom right) indicate 50 μm and 25 μm for the enlarged images

**Fig. 5:**
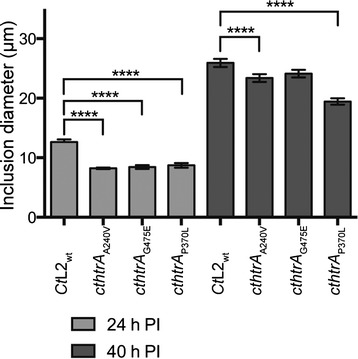
Inclusion sizes of *Ct*L2_wt_ and mutants at 24 h and 44 h post infection. Inclusion sizes were measured from independent coverslips. ****indicates *p* < 0.0001. Error bars represent standard error of the mean (*n* = 27)

The impact of the mutations on the chlamydial inclusion morphology was examined using immunocytochemistry and confocal laser scanning microscopy. Cultures were examined at 24 h post-infection (log phase) and 40 h post-infection (stationary phase; Fig. 4). At 24 h post-infection, the inclusion size of each mutant appeared smaller compared to the wild-type, while in the *cthtrA*_P370L_ strain there appeared to be fewer chlamydial cells within the inclusions. At 40 h post-infection, the inclusion sizes appeared to be more comparable to the wild-type, if slightly smaller. The inclusion sizes were measured to allow statistical comparison of the mutants against the wild-type, confirming that the mutant inclusion sizes were appreciably smaller compared to the wild-type at 24 h post-infection (Fig. 5). The difference was less pronounced at 40 h post-infection, where the *cthtrA*_G475E_ inclusion sizes were not significantly different compared to the wild-type. Alternatively, in the *cthtrA*_A240V_ and, to a greater extent, *cthtrA*_P370L_ strains, inclusion sizes were significantly smaller than the wild-type (*p* < 0.0001; Fig. 5).

### Genomics and lateral gene transfer to isolate chlamydial SNVs for further characterization resulted in a ctl0738_null_ but not a cthtrA_P370L_ isogenic strain

While these observed phenotypes are consistent with our previous observations for *Ct*HtrA function, it is likely that the additional SNVs present in these mutant genomes also contribute to the phenotype. As a result, the genome sequences of the *cthtrA*_A240V_, *cthtrA*_G475E_, and *cthtrA*_P370L_ mutants were determined by whole genome sequencing (WGS). When compared to the *Ct*L2_wt_ genome sequence, the mutants consisted SNVs at nine loci that were consistent in all three isolates (*cthtrA*_A240V_, *cthtrA*_G475E_, and *cthtrA*_P370L_): *ctl0103*, *nusA*, *ctl0518*, *clpC-1*, *clpC-2*, *rpoB*, *pykF*, and *pmpC*, which were confirmed to originate from the *Ct*L2_rif_ strain used to generate the library (Additional file 1: Table S2) and were thus not expected to contribute to the observed phenotypes. Alternatively, we observed 13 unique SNVs in the *cthtrA*_A240V_ (including the A240V C – T transition at position 247526 in *cthtrA*), 19 unique SNVs in the *cthtrA*_P370L_ isolate (including the P370L C – T transition at position 247916 in *cthtrA*), and eight unique SNVs in the *cthtrA*_G475E_ isolate (including the G475E G – A transition at position 248231 in *cthtrA*; Additional file 1: Table S3), which could potentially contribute to the observed phenotypes.

**Fig. 6:**
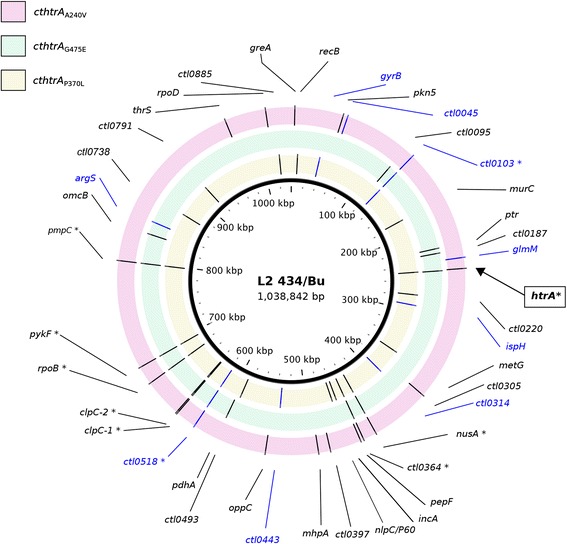
Circular representation of the reference L2/434/Bu genome (1.04 Mbp) showing the position of SNVs found on protein coding genes and the annotation of those genes. Each mutant strain is represented by a single ring layer (from inner to outer: *cthtrA*_P3470L_, *cthtrA*_G475E_, *cthtra*_A240V_). Blue labels correspond to synonymous SNVs and black labels indicate non-synonymous SNVs. Figure generated using BRIG [37]

Given that the isolate with the *cthtrA*_P370L_ mutation displayed the most marked phenotypes and was severely impaired during *Ct*HtrA recombinant protein in vitro protease assays, it was reasoned that this isolate will be the most informative for determining the physiological function of CtHtrA and was selected for further genetic characterization. Of the 19 unique SNVs in the genome of the *cthtrA*_P370L_ mutant isolate, 11 were non-synonymous, five were synonymous, and three were located in intergenic regions. Notably, there was a null mutation in *ctl0738*, a putative DNA methyltransferase. Of the eleven non-synonymous mutations, three resulted in a change to a similar amino acid (i.e. polar, hydrophobic etc.) and are therefore unlikely to have a functional effect (these were SNVs found in *ctl0493*, *met*G, and *ctl0220*). Consequently, a total of eight SNVs in the following *cthtrA*_P370L_ isolate were identified as potentially significant, found on the following locus: *rec*B, *mur*C, *inc*A, *ydhO*, *ctl0738* (putative DNA methyltransferase), *ctl0791* (putative membrane protein), *ctl0885* (conserved hypothetical protein), and *cthtrA*_P370L_ (Fig. 6, Additional file 1: Table S3).

In an effort to separate the *cthtrA*_P370L_ SNV from these remaining SNVs, a lateral gene transfer approach was utilized, by co-infecting host cells with the rifampicin resistant *cthtrA*_P370L_ stain and a spectinomycin resistant *Ct*L2_wt_ strain to generate recombinant isolates [21]. Positive recombinants were selected by plaque purification in the presence of both antibiotics. PCR and Sanger sequencing was conducted on 56 plaque-purified double resistant recombinant strains for the *cthtrA*_P370L_ SNV and *ctl738*_null_ (DNA methyltransferase), in addition to the other SNVs identified as potentially significant. The *cthtrA*_P370L_ SNV was not detected in any of the 56 plaque purified recombinant isolates, while the *ctl0738*_null_ SNV was variously distributed among the recombinants. One recombinant isolate (*ctl0738*_null_) contained the *ctl0738* SNV and none of the other seven SNVs, allowing the potential for characterization of the DNA methyltransferase null mutation in the absence of the *cthtrA*_P370L_ and remaining SNVs.

### Characterization of the ctl0738_null_ mutation suggests that the cthtrA_P370L_ SNV is the major contributor to the reduced infectious progeny phenotype

**Fig. 7:**
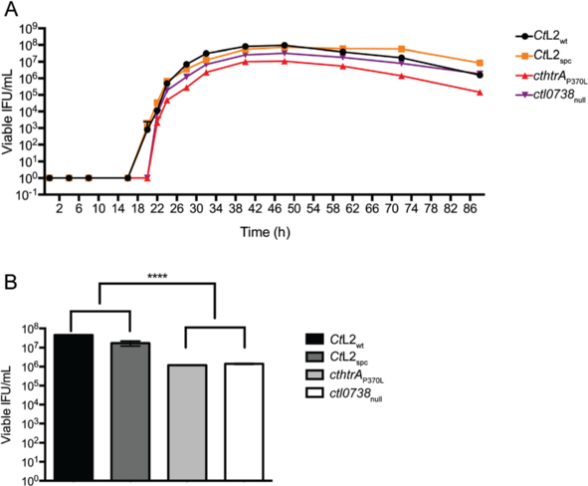
Cell culture analysis of growth and heat shock phenotypes for the *cthtrA*_P370L_ and *ctl0738*_null_ mutants. **a.** One-step growth curve for wild-type *C. trachomatis* L2 and mutants over an 88 h time course. McCoy B cells were infected at an MOI of 0.3 and collected over the time course for determination of inclusion-forming units. **b.** Viable infectious yield of wild-type *C. trachomatis* L2 and mutants at 44 h post infection after 4 h heat shock (42 °C, 5 % CO_2_; 20–24 h post infection). ****indicates *p* < 0.0001. Error bars represent standard error of the mean (*n*–27)

The lateral gene transfer experiments did not enable the generation of an isogenic *cthtrA*_P370L_ mutant isolate. The SNV that could be contributing to the phenotype observed in the *cthtrA*_P370L_ strain was the null mutation in the DNA methyltransferase, *ctl0738*. This mutation was able to be transferred to the genome of *C. trachomatis* L2 spectinomycin-resistant strain (*Ct*L2_spc_) in the absence of the eight other EMS mutations that were considered potentially significant on the *ctHtrA*_P370L_ isolate genome. Therefore, further analysis of the isogenic strain containing the DNA methyltransferase null (*ctl0738*_null_) was conducted to examine the role of this mutation for the phenotypes observed in the *cthtrA*_P370L_ strain. Growth curve and heat shock experiments were conducted using the wild type strains (*Ct*L2_wt_ and *Ct*L2_spc_), the original *cthtrA*_P370L_ mutant strain, and the *ctl0738*_null_ strain. The *cthtrA*_P370L_ strain has the same severely impacted reduced infectious progeny yield (8–25-fold reduction in infectious progeny; Fig. 7a) and the *ctl0738*_null_ strain showed infectious progeny production similar to the wild type. Notably, by including additional time-points at the beginning of the replicative phase (16–22 h post infection), it was observed that both the *cthtrA*_P370L_ and *ctl0738*_null_ strains displayed a delayed start to the log growth phase implying slowed RB to EB conversion. Somewhat unexpectedly, the heat stress phenotype was similar in the *cthtrA*_P370L_ and *ctl0738*_null_ strains with a significant reduction in infectious progeny (*Ct*L2_wt_: 4.5 × 10^7^ EBs, *cthtrA*_P370L_: 1.2 × 10^6^ EBs; *ctl0738*_null_: 1.4 × 10^6^ EBs; *p* < 0.0001), corresponding with a 30–40-fold reduction in infectious progeny after heat stress relative to *Ct*L2_wt_ (Fig. 7b).

## Discussion

Here we present genetic evidence that *cthtrA* has a role in the production of infectious EBs. There are a number of possible conclusions that could be hypothesized based on the results presented here. Firstly, we did not find *cthtrA* alleles with null mutations in either library, so it is tempting to speculate that such a mutation would not be viable, however, considerably greater numbers of mutant strains would need to be screened before we could conclude with confidence that null mutations in *cthtrA* are not permissible. Although, we have previously demonstrated that a *Ct*HtrA specific chemical inhibitor of its protease activity is completely lethal when added during the replicative phase of the developmental cycle [15], which supports that it may not be possible to isolate strains with null mutation in *cthtrA*.

We were able to identify a strain with a SNV resulting in an amino acid substitution (P370L) in the *cthtrA* sequence, that resulted in a severe reduction in proteolytic activity in the recombinant protein bearing the substitution. However, we expect there is some proteolytic activity by this form of *Ct*HtrA (*cthtrA*_P370L_) in vivo. Firstly, our in vitro assays are based on model substrates requiring a second peptide for allosteric activation of maximal protease activity and thus may not accurately reflect in vivo activity. Secondly, the assay against the full-length β-casein protein demonstrated in vitro protease activity for this mutated protein, suggesting that in vivo activity is likely to be present. Given that the strain is still viable and that chemical inhibition of the proteolysis activity of *Ct*HtrA was found to be lethal [15], we suspect some *Ct*HtrA proteolysis activity is occurring in vivo in this strain (*cthtrA*_P370L_). While causality will require additional genetic evidence, this mutation to be a major contributor to the severe defect in the production of infectious EBs we observed in the *cthtrA*_P370L_ mutant strain (20–40-fold reduction), that was not contributed to by the *ctl0738* SNV (as this was not observed in the *ctl0738*_null_ strain generated by lateral gene transfer).

The *cthtrA*_P370L_ strain had 18 SNVS compared to 11 and 8 SNVs detected in the *cthtrA*_A240V_, *cthtrA*_G475E_ strains respectively, and hence, while it is tempting to attribute the phenotypes observed to the P370L mutation, it could in fact be a consequence of a combination of mutations or one of the other mutations alone. However, we can be confident that the reduced infectious EB production phenotype is not a consequence of the *ctl0738*_null_ mutation, based on the data in Fig. 7a. It was surprising to observe that the *ctl0738*_null_ strain showed similar reduced infectious EB yield after heat stress as the *cthtrA*_P370L_ isolate, as this model of stress is not expected to induce DNA damage that would require the repair enzyme predicted to be encoded by this locus [35].

Perhaps the most important result is that after lateral gene transfer experiments and the screening of 56 plaque purified isolates, no strain was identified containing the P370L mutation (C – T at nucleotide position 247916). This could be random chance or could suggest that the P370L mutation in isolation is responsible for a severe growth defect that prevented us from isolating any strains during these experiments. Alternatively, it is possible that one of the other SNVs is suppressing the P370L mutation in the *cthtrA*_P370L_ strain. Given that we expect *Ct*HtrA’s function to impact the integrity of several chlamydial cell envelope proteins, including major outer membrane protein (MOMP), Porin B (PorB), and the polymorphic membrane proteins (Pmps) [12], *Ct*HtrA’s chaperone and protease activity as a stress response and housekeeping protein important for outer membrane protein assembly could be indirectly driving the observed phenotypes [31, 36].

The smaller inclusion sizes observed in the *cthtrA*_P370L_ strain corresponds with the reduced infectious EB yield suggesting a growth defect (possibly relating to optimal replication) could be a consequence of reduced *Ct*HtrA function, likely indirectly due to the function of a substrate that requires *Ct*HtrA for correct assembly or maintenance.

Overall, we have provided genetic evidence that *C. trachomatis* with a genetic mutation (C – T at position 247916 in *cthtrA*) likely resulted in reduced production of infectious EB, potentially correlating with a negative impact of this mutation on *Ct*HtrA’s in vitro proteolytic activity. While genetic causality could not be confirmed, these results provide further evidence that *Ct*HtrA has a critical role during the developmental cycle of *Chlamydia*.

## Conclusions

HtrA is known to be an important protease for many bacterial pathogens.  Analysis of Chlamydia trachomatis genetic mutants possessing variants of the *cthtrA* gene that firstly showed an impact on proteolytic function by in vitro analysis demonstrated that these mutants had reduced infectious progeny. Thus HtrA has a critical role in formation of chlamydial infectious progeny.

### Availability of supporting data

Supplementary tables are provided. The draft assemblies of all genome sequences generated during this project are available through the EMBL ENA database under the Study number PRJEB9044: http://www.ebi.ac.uk/ena/data/search?query=PRJEB9044.

## Additional files

Additional file 1: Table S1. EMS library mutants with confirmed non-synonymous SNVs in the *cthtrA* gene. Table S2. List of bacterial strains used in this study. **Table S3.** Total SNVs present in the *cthtrA*_A240V_, *cthtrA*_P370L_, and *cthtrA*_G475E_ mutants as confirmed by whole genome sequencing. **Table S4.** Sequences and associated annealing temperatures for the PCR primers used in this study. Table S5. Active site substrates and activators used for proteolysis and oligomerisation assays. (DOCX 45 kb) Additional file 2: Figure S1. SDS-PAGE gels of full length β-casein cleavage by wild type *Ct*HtrA and mutants over a 60 min time course. A. Wild-type *Ct*HtrA; B. *Ct*HtrA_A240V_; C. *Ct*HtrA_E47K_; D. *Ct*HtrA_G268R_; E. *Ct*HtrA_G475E_; F. *Ct*HtrA_P370L_; G. *Ct*HtrA_R55Q_. Lanes are labelled: M: protein molecular weight marker (Bio-Rad); 0 min; 10 min; 20 min; 30 min; 40 min; 50 min; 60 min; H: *Ct*HtrA only; βc: β-casein only. The molecular masses of standard proteins are indicated by arrows next to the gels. (DOC 21 kb)

## Abbreviations

DNP: 2,4-dinitrophenyl
EB: Elementary body
EMS: Ethyl methanesulfonate
FCS: Fetal calf serum
MCA: 7-methoxycoumarin-4-acetic acid
MOMP: Major outer membrane protein
OMP: Outer membrane protein
Pmp: Polymorphic membrane protein
*p*NA: *para*-Nitroanilide
RB: Reticulate body
Rif^R^: Reifampicin-resistant
SNV: Single nucleotide variant
Spc^R^: Spectinomycin-resistant
TILLING: Targeting induced local lesions in genomes
WGS: Whole genome sequencing
