# Supplementary material for: A Chlamydia trachomatis strain with a chemically generated amino acid substitution (P370L) in the cthtrA gene shows reduced elementary body production
Source: BMC Microbiol. 2015 Sep 30;15:194. doi: 10.1186/s12866-015-0533-2 (PMC4590699; doi:10.1186/s12866-015-0533-2)
Supplement: Additional file 1: Table S1. — EMS library mutants with confirmed non-synonymous SNVs in the cthtrA gene. Table S2. List of bacterial strains used in this study. Table S3. Total SNVs present in the cthtrA A240V, cthtrA P370L, and cthtrA G475E mutants as confirmed by whole genome sequencing. Table S4. Sequences and associated annealing temperatures for the PCR primers used in this study. Table S5. Active site substrates and activators used for proteolysis and oligomerisation assays. (DOCX 45 kb) [file 12866_2015_533_MOESM1_ESM.docx]

**Supplementary Tables**

**Supplementary Table 1. EMS library mutants with confirmed non-synonymous SNVs in the *cthtrA* gene.**

| **Mutants** | **Domain** | **Structural loops** | **EcHtrA** | **Potential significance based on models** |
| --- | --- | --- | --- | --- |
| A24T | Signal peptide | N/A | Leu07 | *Not in model* |
| G40R | Signal peptide | N/A | Ala09 | *Not in model* |
| E43K | Protease | N/A | Met12 | Minor to none |
| E47K | Protease | α1 | Ala16 | Potential steric clash with adjacent protease domain |
| R55Q | Protease | α1 | Pro24 | Disruption of salt-bridge with D157 of loop LE of adjacent protease domain |
| P66L | Protease | β1 | Thr35 | Minor to none |
| V69I | Protease | β1 | N/A | Minor to none |
| G78R | Protease | Loop LA | Pro43 | Disruption of LA loop beta strands |
| S84F | Protease | Loop LA | Phe49 | Disruption of LA:LA loop interaction |
| P85S | Protease | Loop LA | Phe50 | Minor to none |
| R89K | Protease | Loop LA | Ser54 | Minor to none |
| D101N | Protease | Loop LA | Ser66 | *Not in model* |
| R118H | Protease | Loop LA | Gln80 | *Not in model* |
| R126L | Protease | β2 | Leu87 | Minor to none |
| G149R | Protease | Loop LB | Thr111 | Potential steric clash with protease domain loop |
| H156Y | Protease | Loop LE | Ser118 | Possible steric interruption to loop L3 |
| T162I | Protease | β5 | Asp124 | Minor to none |
| H228Y | Protease | Loop L3 | Asn191 | Steric clash with adjacent protease domain |
| A240V | Protease | Loop L1 | Ala203 | Disruption of loop L1 conformation and active site serine |
| G268R | Protease | Loop L2 | Pro231 | Disruption of loop L2 conformation and polar interaction with S267 (L2); clash with L2 of adjacent protease domain |
| R286Q | Protease | α1 | Asn249 | Minor to none |
| V304I | PDZ1 | Carboxylate binding loop | Ile267 | May impact substrate specificity |
| V330I | PDZ1 | β14 | Val293 | May contribute to PDZ1:PDZ2 interaction but V:I mutation redundant |
| A360V | PDZ1 | PDZ1 activation cleft | Ala323 | Minor to no noticeable disruption - does not contribute to hydrophobic cleft |
| P370L | PDZ1 | β16 | Val333 | Potential steric clash with protease - PDZ1 loop; may impact substrate specificity |
| V379I | PDZ1 | β16 | Leu342 | Minor to none |
| G382R | PDZ1 | β16 | Gly345 | Minor to none |
| G382E | PDZ1 | β16 | Gly345 | Minor to none |
| P415S | PDZ2 | β18 | Lys381 | Disruption to PDZ2 loop |
| A442T | PDZ2 | α10 | Ala397 | Minor to none |
| S443F | PDZ2 | α10 | Gln398 | Disruption to adjacent PDZ2 loop |
| V446I | PDZ2 | α10 | Leu401 | Minor to none |
| P448S | PDZ2 | α10 | Lys403 | Disruption to PDZ2 loop |
| V455M | PDZ2 | β20 | Ala410 | Disruption to adjacent beta strand |
| G475E | PDZ2 | α11 | Pro429 | Potential disruption of two PDZ2 loops |

This table lists all EMS-treated chlamydial isolates containing a single non-synonymous SNV in the *cthtrA* gene. The mutants (column 1) are the specific *Ct*HtrA amino acid position and mutations. The domain and structural loop columns indicate the mutation position according to the *Ct*HtrA secondary and tertiary structures predicted by homology modeling. The *Ec*HtrA column lists the corresponding amino acid and position in *E. coli* DegP. The potential significance of these mutations were predicted according to the *Ct*HtrA homology models. The six most significant mutants, based on their predicted functional impact, are highlighted in blue.

**Supplementary Table 2. List of bacterial strains used in this study.**

| **Strain** | **Parental strain** | **Accession no.** | **Genotype*** | **Ref.** |
| --- | --- | --- | --- | --- |
| *Ct*L2_wt_ | 434/Bu LGV L2 | PRJEB9044 | *CTL0259_1* | [This paper] |
| *Ct*L2_rif_ | 434/Bu LGV L2 | N/A | *CTL0103_1 nusA1 CTL0364_1 CTL0518_1 clpC1-1 clpC1-2 rpoB1 pykF1 pmpC1* | [1] |
| *Ct*L2_spc_ | 434/Bu LGV L2 | N/A | *CTL0103_1 CTL_r01_2 CTL_r02_2* | [1] |
| *cthtrA*_A240V_ | *Ct*L2_rif_ | PRJEB9044 | *greA1 pkn5_1 htrA1 CTL0305_1 pepF1 CTL0397_1 mhpA1 oppC1 thrS1* | [2] |
| *cthtrA*_G475E_ | *Ct*L2_rif_ | PRJEB9044 | *CTL0995_1 ptr1 CTL0178_1 htrA1 pdhA1 omcB1 argS1* | [2] |
| *cthtrA*_P370L_ | *Ct*L2_rif_ | PRJEB9044 | *recB1 murC1 htrA1 CTL0220_1 ispH1 metG1 incA1 nlpC/P60_1 CTL0493_1 CTL0738_1 CTL0791_1 CTL0885_1* | [2] |
| *ctl0738*_null_ | *cthtrA*_P370L_ × *Ct*L2_spc_ | PRJEB9044 | *CTL_r01_2 CTL_r02_2 CTL0518_1 clpC1-1 clpC1-2 rpoB1 pykF1 pmpC1 CTL0738_1* | [This paper] |
| *Ct*D-LC | D/UW-3/CX | CP002054 | *ffh1 CT049_1 CT135_1 CT352_1 hrcA1 rl15_1 dacC1 cpxR1 CT638_1 recC1* | [3] |

***** The genotype indicates the locus where SNVs were identified when compared to the *C. trachomatis* L2 434/Bu reference genome (NC_010287.1).

**Supplementary Table 3. Total SNVs present in the *cthtrA*_A240V_, *cthtrA*_P370L_, and *cthtrA*_G475E_ mutants as confirmed by whole genome sequencing.**

| **No.** | **Locus** | **Position** | **Gene** | **Product** | **Ref** | **Alt** | **AA** | | **S/NS** | | **Notes** |
| --- | --- | --- | --- | --- | --- | --- | --- | --- | --- | --- | --- |
| **Unique *cthtrA*_A240V_ SNVs** | | | | | | | | | | | |
| 1 | CTL0004 | 3442 | *greA* | Transcription elongation factor | G | A | Gly-Ser | | NS | |  |
| 2 | CTL0042 | 50241 | *pkn5* | putative serine/threonine-protein kinase (TTS effector) | C | T | Arg-Trp | | NS | |  |
| 3 | CTL0045 | 54667 |  | conserved hypothetical protein | G | A | Leu-Leu | | S | |  |
| 4 | CTL0187 | 236971 |  | phosphoglucosamine mutase | G | A | Val-Val | | S | |  |
| 5 | CTL0195 | 247526 | *htrA* | serine protease | C | T | Ala-Val | | NS | | A240V |
| 6 | CTL0305 | 377808 |  | conserved hypothetical protein | G | A | Gly-Ser | | NS | |  |
| 7 | CTL0367 | 451644 | *pepF* | oligoendopeptidase F | G | A | Thr-Ile | | NS | |  |
| 8 | CTL0397 | 481835 |  | conserved hypothetical protein | C | T | Ser-Leu | | NS | |  |
| 9 | CTL0403 | 493268 | *mhpA* | FAD-dependent monooxygenase | G | A | Ser-Phe | | NS | |  |
| 10 | CTL0452 | 544242 | *oppC* | oligopeptide transport system membrane permease | C | T | Pro-Ser | | NS | |  |
| 11 | CTL0844 | 974393 | *thrS* | threonyl-tRNA synthetase | G | A | Asp-Asn | | NS | |  |
| 12 | CTL0879 | 1014167 | *rpoD* | RNA polymerase sigma factor | C | T | Asp-Asn | | NS | |  |
| **Unique *cthtrA*_P370L_ SNVs** | | | | | | | | | | | |
| 1 | CTL0007 | 8248 | *recB* | exodeoxyribonuclease V beta chain | G | A | Ser - Phe | | NS | |  |
| 2 | CTL0030 | 37611 | *gyrB* | DNA gyrase subunit B | G | A | Gly - Gly | | S | |  |
| 3 | intergene | 123163 |  | upstream of *ribA* | C | A | N/A | | N/A | | b/t *ribD* + *ribA* |
| 4 | CTL0131 | 176538 | *murC* | UDP-N-acetylmuramate--alanine ligase | C | T | Pro - Ser | | NS | |  |
| 5 | CTL0195 | 247916 | *htrA* | serine protease | C | T | Pro - Leu | | NS | | P370L |
| 6 | CTL0220 | 277919 |  | conserved hypothetical protein | C | T | Met - Ile | | NS | |  |
| 7 | CTL0234 | 291886 | *ispH* | 4-hydroxy-3-methylbut-2-enyl diphosphate reductase | C | T | Ile - Ile | | S | |  |
| 8 | CTL0287 | 357982 | *metG* | methionyl-tRNA synthetase | G | A | Met - Ile | | NS | |  |
| 9 | CTL0314 | 390141 |  | putative membrane protein | G | A | Ala - Ala | | S | |  |
| 10 | CTL0374 | 459480 | *incA* | inclusion membrane protein A | C | T | Gly - Arg | | NS | |  |
| 11 | CTL0382 | 465024 | *nlpC/P60* | chitinase | C | T | Gly - Arg | | NS | |  |
| 12 | CTL0443 | 533829 |  | conserved hypothetical protein | G | A | Pro - Pro | | S | |  |
| 13 | CTL0493 | 587766 |  | outer membrane protein (variable surface antigen) | G | A | Val - Ile | | NS | |  |
| 14 | intergene | 734661 |  | upstream of dapB | C | T | N/A | | N/A | | b/t dapB + CTL619 |
| 15 | CTL0738 | 870646 | *ada* | putative DNA methyltransferase | C | T | Gln - * | | NS | |  |
| 16 | CTL0791 | 916268 |  | putative membrane protein | C | T | Ala - Thr | | NS | |  |
| 17 | intergene | 929717 |  | upstream of uhpC | C | A | N/A | | N/A | | b/t hisS + uhpC |
| 18 | CTL0885 | 1023181 |  | conserved hypothetical protein | C | T | Gly - Arg | | NS | |  |
| **Unique *cthtrA*_G475E_ SNVs** | | | | | | | | | | | |
| 1 | CTL0095 | 117642 |  | cell cycle protein | G | A | Pro-Ser | NS | |  | |
| 2 | CTL0175 | 223227 | *ptr* | exported insulinase/protease | G | A | Ala-Val | NS | |  | |
| 3 | CTL0178 | 228634 |  | conserved hypothetical protein | G | A | Asp-Asn | NS | |  | |
| 4 | CTL0195 | 248231 | *htrA* | serine protease | G | A | Gly-Glut | NS | | G475E | |
| 5 | CTL0497 | 592994 | *pdhA* | pyruvate dehydrogenase E1 component alpha | C | T | Pro-Leu | NS | |  | |
| 6 | CTL0702 | 832163 | *omcB* | 60 kD cysteine-rich outer membrane protein | C | T | Val-Met | NS | |  | |
| 7 | CTL0714 | 846248 | *argS* | arginyl-tRNA synthetase | C | T | Leu-Leu | S | |  | |

This table lists each SNV identified in the *cthtrA*_A240V_, *cthtrA*_G475E_, and *cthtrA*_P370L_ isolates by whole genome sequencing, compared to the *Ct*L2_wt_ strain. The final eight SNVs in the table are those that are present in all three mutant isolates. Each SNV is numbered and the gene locus and SNV position is presented according to the *C. trachomatis* L2/434/Bu reference genome (GenBank no. NC_010287). The gene name and product are listed according to the reference genome annotations. The Ref and Alt columns indicate the specific nucleotide change according to the reference genome and mutant genome, respectively. The corresponding amino acid changes and their classification as synonymous or non-synonymous mutations are listed, in addition to relevant notes relating to select SNVs. The most significant non-synonymous SNVs in the *cthtrA*_P370L_ isolate are highlighted in blue.

**Supplementary Table 4. Sequences and associated annealing temperatures for the PCR primers used in this study.**

| **Primer** | **Primers** | **T_A_** |
| --- | --- | --- |
| A240Vsdm | FOR: 5’ – ACTTTATTCAAACAGATGTTGCCATTAATCCTGGGAATTC – 3’ | 72ºC |
|  | REV: 5’ – GAATTCCCAGGATTAATGGCAACATCTGTTTGAATAAAGT – 3’ |  |
| E47Ksdm | FOR: 5’ – AAGAGGTTTCACAAAAAGATCTGCTCAAAGAAGTATCC – 3’ | 72ºC |
|  | REV: 5’ – GGATACTTCTTTGAGCAGATCTTTTTGTGAAACCTCTT – 3’ |  |
| G268Rsdm | FOR: 5’ – AATACTGCCATCGTCAGTCGTAGCGGGGGATATATTGG – 3’ | 77ºC |
|  | REV: 5’ – CCAATATATCCCCCGCTACGACTGACGATGGCAGTATT – 3’ |  |
| G475Esdm | FOR: 5’ – TGAAAAACTCGAAAGAAGAGAATGTTCTCCTTATGGTTTC – 3’ | 72ºC |
|  | REV: 5’ – GAAACCATAAGGAGAACATTCTCTTCTTTCGAGTTTTTCA – 3’ |  |
| P370Lsdm | FOR: 5’ – ATTTCCCTAATGATGCTAGGGACTCGTGTTATTTTAA – 3’ | 71ºC |
|  | REV: 5’ – TTAAAATAACACGAGTCCCTAGCATCATTAGGGAAAT – 3’ |  |
| R55Qsdm | FOR: 5’ – TGCTCAAAGAAGTATCCCAAGGATTTTCTCGGGTCGC – 3’ | 77ºC |
|  | REV: 5’ – GCGACCCGAGAAAATCCTTGGGATACTTCTTTGAGCA – 3’ |  |

**Supplementary Table 5. Active site substrates and activators used for proteolysis and oligomerisation assays.**

| **I.D.** | **Sequence** | **Function** |
| --- | --- | --- |
| βcas1 | MCA-ENLHLPLPIIF-DNP | Substrate |
| pNA1 | DPMFKLV-pNA | Substrate |
| pNA2 | PMFKLI-pNA | Substrate |
| pNA3 | MFKLI-pNA | Substrate |
| pNA4 | MFQLI-pNA | Substrate |
| β-casein | [full-length protein] | Both |
| Act1 | NH_2_-VLGPVRGPFPIIF-OH | Activator |

**Supplementary References**

1. Nguyen BD, Valdivia RH: **Virulence determinants in the obligate intracellular pathogen *Chlamydia trachomatis* revealed by forward genetic approaches.** *Proc Natl Acad Sci USA* 2012, **109**:1263–1268.

2. Snavely EA, Kokes M, Dunn JD, Saka HA: **Reassessing the role of the secreted protease CPAF in *Chlamydia trachomatis* infection through genetic approaches**. *Pathog Dis* 2014, **0**:1–16.

3. Sturdevant GL, Kari L, Gardner DJ, Olivares-Zavaleta N, Randall LB, Whitmire WM, Carlson JH, Goheen MM, Selleck EM, Martens C, Caldwell HD: **Frameshift mutations in a single novel virulence factor alter the *in vivo* pathogenicity of *Chlamydia trachomatis* for the female murine genital tract.** *Infect Immun* 2010, **78**:3660–3668.
