# Supplementary figures and images for: A Chlamydia trachomatis strain with a chemically generated amino acid substitution (P370L) in the cthtrA gene shows reduced elementary body production
Source: BMC Microbiol. 2015 Sep 30;15:194. doi: 10.1186/s12866-015-0533-2 (PMC4590699; doi:10.1186/s12866-015-0533-2)

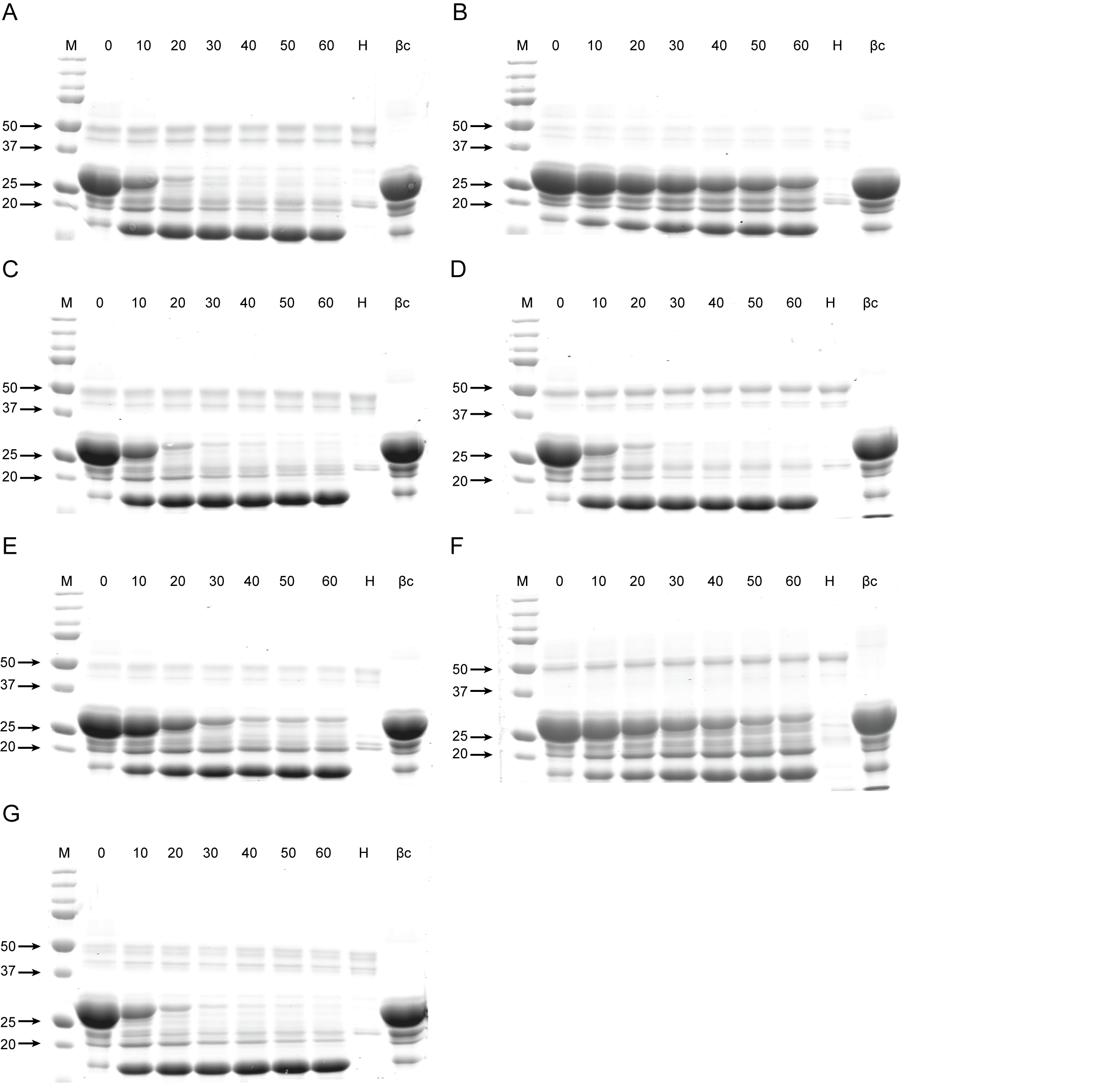

Supplement: Additional file 2: Figure S1. — SDS-PAGE gels of full length β-casein cleavage by wild type CtHtrA and mutants over a 60 min time course. A. Wild-type CtHtrA; B. CtHtrAA240V; C. CtHtrAE47K; D. CtHtrAG268R; E. CtHtrAG475E; F. CtHtrAP370L; G. CtHtrAR55Q. Lanes are labelled: M: protein molecular weight marker (Bio-Rad); 0 min; 10 min; 20 min; 30 min; 40 min; 50 min; 60 min; H: CtHtrA only; βc: β-casein only. The molecular masses of standard proteins are indicated by arrows next to the gels. (DOC 21 kb) [file 12866_2015_533_MOESM2_ESM.doc]
